# Hybrid exosome–liposome nanovesicles as delivery platforms for therapeutic applications: merging the best of both worlds

**DOI:** 10.7150/thno.135691

**Published:** 2026-09-18

**Authors:** Jian Yu, Lujiao Yu, Fei Ren, Kai Cui, Gongping Sun, Hong Pan

**Affiliations:** 1 Department of Neurosurgery, The Fourth Affiliated Hospital of China Medical University, No. 4 Chongshandong Road, Shenyang, 110032, China; 2 Department of Geriatrics, The First Hospital of China Medical University, No. 155 North Nanjing Street, Shenyang, 110001, China; 3 Department of Orthopaedics, The Fourth Affiliated Hospital of China Medical University, No. 4 Chongshandong Road, Shenyang, 110032, China; 4 Department of General Surgery, The Fourth Affiliated Hospital of China Medical University, No. 4 Chongshandong Road, Shenyang, 110032, China; 5 Department of Radiation Oncology, The Fourth Affiliated Hospital of China Medical University, No. 4 Chongshandong Road, Shenyang, 110032, China

**Keywords:** exosome, liposome, nanovesicle, drug delivery, membrane fusion

## Abstract

Exosomes are increasingly employed to treat various diseases because of their remarkable ability to enhance the stability of encapsulated cargoes. This capability is attributed to their outstanding homologous targeting, desirable biocompatibility, and low immunogenicity, thereby contributing to improved therapeutic outcomes. Nevertheless, exosomes are constrained by some limitations, including poor loading capacity, inherent heterogeneity, and challenges in large-scale production. Consequently, hybrid exosome-liposome (EL) systems, which can circumvent these limitations, are playing an increasingly crucial role in therapeutic applications. Herein, we introduce the two primary strategies for drug loading into exosomes -- passive loading and active loading. We further elaborate on the fabrication methods of hybrid ELs and their applications in drug delivery, highlighting the innate advantages of these state-of-the-art nanovesicles over conventional exosomes and liposomes. Major challenges and future perspectives of hybrid ELs in treating various diseases are also discussed.

## 1. Introduction

Exosomes are nanovesicles, sized 30–200 nm, that are naturally secreted by diverse cells, including immune cells, stem cells, and tumor cells 1,2. These nanovesicles carry cell-specific molecules, including tetraspanin proteins and functional nucleic acids, and thus play a key role in intercellular communication and physiological regulation 3,4. In addition, exosomes are widely present in biofluids, such as blood, urine, and cerebrospinal fluid 5. Exosomes are also found in edible organisms, including plants 6 and milk 7. Furthermore, exosomes possess innate abilities to traverse physiological barriers, such as the blood-brain barrier (BBB) 8, blood-retinal barrier (BRB) 9, and blood-placental barrier 10, making them unique vehicles. Despite these advantages, exosomes face critical challenges in achieving desirable cargo payloads 11,12. These challenges stem from limited loading capacity and a complex fabrication process 13,14.

Liposomes are artificial lipid bilayer-based vesicles 15,16. They can overcome the limitations of exosomes by offering efficient drug loading and scalable manufacturing 17, 18. In addition, liposomes can be easily modified during fabrication, such as PEGylation, targeting ligand conjugation, and incorporation of environmentally responsive lipids 19,20. However, liposomes lack endogenous targeting ligands, resulting in non-specific biodistribution and rapid systemic clearance by MPS 21,22. In contrast, exosomes show innate advantages, including homologous targeting, prolonged circulation, and low immunogenicity 23-25.

Intriguingly, the strengths of exosomes and liposomes complement each other. Hybrid exosome-liposome (hybrid EL) systems have received increasing attention for drug delivery, because they combine the biological recognition and prolonged circulation of exosomes with the payload flexibility and production reproducibility of liposomes 26,27. Hybrid ELs can eliminate the need for targeted liposomes for exogenous ligands by using surface proteins of exosomes for targeted delivery 28,29. For instance, hybrid ELs show improved tumor accumulation compared with conventional liposomes, by utilizing exosomal adhesion molecules to facilitate active targeting 30,31. Moreover, hybrid ELs mitigate the immunogenicity risks of synthetic carriers and retain the ability to traverse physiological barriers 32,33. The incorporation of liposomal components also helps overcome the limited loading capacity and low production yields of exosomes 34,35. Collectively, exosomes and liposomes can “learn” from each other, and hybrid ELs that integrate the biological advantages of exosomes with the engineering adaptability of liposomes are rapidly emerging.

Although several reviews have discussed hybrid ELs, few have focused primarily on their therapeutic applications, and detailed elaboration on their use in drug delivery remains limited 36-40. This review summarizes the advantages and disadvantages of liposomes and exosomes separately. As illustrated in Figure 1, the fabrication methods and diverse loading strategies for hybrid EL cargoes are also discussed. Moreover, the applications of these state-of-the-art nanovesicles for drug delivery are elaborated upon.

## 2. Liposomes for drug delivery

Liposomes are a classic drug delivery system with various advantages, such as high loading efficiency, minimal adverse effects, and ease of fabrication and modification 17,41. Both hydrophilic and lipophilic drugs can be loaded in liposomes. Hydrophilic drugs, such as mRNA 42, miRNA 43, and siRNA 44, can be entrapped in aqueous lumens of liposomes. In contrast, lipophilic drugs can be inserted into the lipid bilayer 45. Various nanoparticles can also be entrapped within liposomes, improving nanoparticle biocompatibility and prolonging circulation time 46,47. For instance, upconversion nanoparticles (UCNPs) were incorporated into membrane-integrated liposomes, enhancing luminescence, improving cellular imaging, and enabling targeted delivery 48. Consequently, liposomes are widely used for co-loading diverse cargos as multifunctional carriers to achieve therapeutic synergy 49, 50. Liposomes also offer convenient industrial-scale manufacturing, with several liposomal products, such as Doxil^®^ (doxorubicin), Lipusu^®^ (paclitaxel), Depocyt^®^ (cytarabine), and Ambisome^®^ (amphotericin B), already in clinical use.

There are two primary strategies to functionalize liposomes: surface modification and composition design. Surface modification involves conjugating functional molecules to the liposomal surface 16, 51. PEGylation is a common strategy to prolong circulation time by forming a hydrophilic layer on the liposomal surface, which reduces plasma protein absorption and opsonization 52. Additionally, targeting ligands, including peptides 53, proteins 54, and aptamers 15, can also be anchored to the liposomal surface to fabricate targeted liposomes. For instance, Luo et al. developed Tet1 peptide-modified liposomes (Lipo-T) for retinal ganglion cell (RGC)-targeting 55. The Tet1 peptide exhibits high affinity for GT1b, which is enriched on RGCs but minimally expressed on glial cells, enabling precise targeting. Tet1 modification peptide enhanced RGC uptake, reduced glial cell activation, and minimized inflammatory responses and adverse side effects. Moreover, Tet1 peptide modification maintained stable drug encapsulation, controlled drug release, improved drug bioavailability, and enabled lower therapeutic doses, thereby reducing systemic toxicity.

During liposome fabrication, functional lipids, including pH-responsive 56, thermo-responsive 57, and ROS-responsive lipids 58, can be incorporated into the lipid bilayer of liposomes. For example, Gao and co-workers synthesized a ROS-responsive lipid, iDAPC, and used it to fabricate ROS-responsive liposomes loaded with the immunostimulatory CpG oligodeoxynucleotides (ODNs). Upon ROS exposure, DAPC underwent structural changes that destabilized ROS-responsive liposomes and selectively released CpG ODNs 58. These ROS-responsive liposomes preferentially released CPG ODNs in malignant cells, promoting dendritic cell maturation and subsequent activation of CD8^+^ T cells. Thus, functionalized liposomes can achieve targeted delivery and prolonged circulation. Nevertheless, targeted liposomes still face challenges, such as non-specific biodistribution, limited circulation time, and potential immunogenicity 59.

## 3. Exosomes for drug delivery

Exosomes are rich in various biomolecules, including proteins, nucleic acids, and metabolites 60, 61. As intercellular messengers, exosomes play a key role in delivering biomolecules and regulating physiological functions in recipient cells 62. As listed in Table 1, exosomes originate from diverse sources that show cell specificity and innate homing capabilities 63. Some exosomal biomarkers include tetraspanins (CD9, CD63, CD81), Alg2-interacting protein X (ALIX), and tumor susceptibility gene 101 (TSG101) 64. Negative control biomarkers, including calnexin, cytochrome c oxidase subunit IV (COX IV), and histone H3, are associated with the endoplasmic reticulum, mitochondria, and cellular nucleus, separately 65. Drug loading into exosomes generally follows two principal strategies: passive loading and active loading 66,67.

### 3.1. Passive loading

Passive loading relies on physicochemical interactions between exosomes and cargos 68. Hydrophobic molecules predominantly partition into the lipid bilayer of exosomes, leading to moderate loading efficiencies 69,70. A major merit is the preservation of membrane integrity, which keeps native surface proteins, such as tetraspanins and integrins 30,71. Despite its simplicity, passive loading often requires substantial quantities of drugs, making purification and optimization challenging 30.

### 3.2. Active loading

Active loading overcomes diffusion limitations, including electroporation, sonication, and freeze-thaw cycles 72,73. Electroporation transiently permeabilizes the exosomal membrane, facilitating nucleic acid encapsulation, although improper conditions may induce cargo aggregation and exosome clumping 28,74. Sonication enhances drug loading by membrane permeabilization, but localized heat can increase the risk of protein denaturation 5,75. Freeze-thaw cycles improve loading ratios of hydrophobic cargoes and preserve exosomal morphology 76. Collectively, active loading facilitates precise loading of diverse cargoes, including small molecules, nucleic acids, and gene-editing systems, but may compromise membrane integrity, accelerate blood clearance, and damage surface proteins 35,77. Thus, passive loading keeps biological function but limits loading efficiency, whereas active loading increases loading efficiency at the cost of exosomal performance 74,78.

## 4. Strategies to fabricate hybrid EL

Though exosomes possess innate biocompatibility and homing capabilities, they face some challenges, such as limited loading capacity and complex fabrication processes 13,14. Their natural composition supports immune evasion and physiological barrier penetration, but leads to variability in production yield and functional consistency 79,80. Conversely, liposomes offer efficient drug loading and scalable manufacturing, yet lack endogenous targeting ligands 5,17,18. Hybrid ELs integrate the strength of both systems – combining exosomal targeting and prolonged circulation with liposomal flexibility and high loading capacity. Three primary strategies to fabricate hybrid EL: co-incubation, freeze-thaw, and co-extrusion, listed in Table 2.

### 4.1. Co-incubation method

The co-incubation method enables membrane fusion between exosomes and liposomes by passive intermolecular interactions, allowing hybridization without compromising structural integrity or bioactivity 81. This method requires no specialized conditions, keeps exosomal surface markers, and integrates liposomal loading capabilities under physiologically compatible environments 82. Though fusion efficiency remains less than that achieved by mechanical or chemical methods, co-incubation minimizes disruptive forces, and is well-suited for keeping labile drugs 30. However, hybrid ELs acquired by co-incubation may show increased particle size and size distributions 83. Optimization of lipid composition and incubation parameters can enhance the stability and functionality of hybrid ELs.

### 4.2. Freeze-thaw method

Freeze-thaw cycles induce membrane fusion by repeated disruption and reformation of membranes, providing an efficient integration of exosome and liposome 84. This method is based on the concept that large vesicles break into smaller ones during freeze-thaw cycles, which promotes fusion 85. Reported fusion efficiencies reach up to 97.4% 86. The process of fabricating freeze-thaw based hybrid ELs keeps the exosomal surface markers essential for targeting and uses the loading capacity of liposomes 87. However, repeated freeze-thaw cycles may compromise membrane integrity or cause cargo leakage, necessitating a balance between fusion efficiency and vesicle stability 88.

### 4.3. Co-extrusion method

The co-extrusion method is widely used to prepare hybrid ELs by mechanically fusing exosomes and liposomes by repeated extrusion across porous filters, producing uniform nanovesicles 89. Shear forces fragment nanovesicles into membrane sheets, which subsequently self-assemble into spherical nanovesicles, a process that reduces the free energy 90. Membrane fusion structures of hybrid EL could be verified by a decline in the Forster resonance energy transfer (FRET) (Figure 2A), due to the membrane fusion of liposomes and exosomes 91. The membrane fusion process was further confirmed by zeta drift (Figure 2B) and retention of platelet-specific markers, such as CD41/P-selectin (Figure 2C).

Co-extrusion enhances drug-loading efficiency of exosomes by utilizing liposomal loading capacity 92. Hybrid ELs fabricated by co-extrusion inherit environmental responsiveness from specialized liposomes and retain exosomal properties that improve targeted accumulation compared with conventional liposomes 89,93. These hybrid ELs also demonstrate superior stability and prolonged circulation compared with co-incubation and freeze-thaw counterparts 94. Optimal extrusion parameters, including pore size, pressure, and number, are essential to prevent excessive membrane disruption and preserve exosomal surface proteins 95,96. For hydrophilic cargos, co-extrusion may yield lower encapsulation efficiency due to concentration constraints during extrusion 97.

## 5. Hybrid exosome-liposome for cargo delivery

### 5.1. Chemotherapeutics delivery

Hybrid ELs combine the biocompatibility and targeting properties of exosomes with the high loading capacity of liposomes, making them highly effective carriers for the delivery of chemotherapeutics. Importantly, these hybrid ELs can enhance therapeutic efficacy while reducing off-target toxicity. For example, doxorubicin (DOX), a widely used anti-cancer drug, suffers from non-specific biodistribution and dose-limiting toxicities, resulting in limited therapeutic efficacy 68,98,99. Hybrid ELs offer a promising strategy to overcome these limitations.

Rayamajhi et al. developed a DOX-loaded hybrid EL by co-extruding macrophage-derived exosomes and pH-sensitive liposomes 83. The DOX-loaded hybrid EL retained the innate targeting properties of macrophage-derived exosomes and the stability and high drug-loading capacity of liposomes. Compared with naive macrophage-derived exosomes, the DOX-loaded hybrid ELs improved homogeneity and colloidal stability, exhibited pH-sensitive drug release in acidic tumor environments, and facilitated efficient cellular uptake. Moreover, the DOX-loaded hybrid ELs exhibited potent cytotoxicity against cancer cells while sparing normal cells, demonstrating their potential for tumor-selective delivery and reduced off-target toxicity.

In another study, tumor-derived exosomes and liposomes were fused to generate DOX-loaded biomimetic nanovesicles (DOX@LINV) for cancer immunotherapy 27. DOX@LINV achieved an 82% tumor suppression rate at a 4-fold reduced DOX dose required for conventional DOX-loaded liposomes (79% suppression), significantly reducing systemic toxicity. Furthermore, DOX@LINV preserved tumor antigens and danger signals, promoting dendritic cell maturation and immunogenic cell death (ICD). Of note, DOX-induced ICD and exosome-mediated immune activation together led to a potent anti-tumor response, including increased T-cell infiltration and modulation of the tumor immunosuppressive microenvironment. These findings highlight the versatility of hybrid ELs in improving chemotherapeutic outcomes and enabling synergistic chemo-immunotherapy.

Plant-derived exosomes have also been explored for drug delivery 5,69. For instance, balloon flower root-derived exosome-like nanoparticles (BDEs), which were isolated by TFF, were co-extruded with silymarin (SM)-loaded liposomes to mitigate acetaminophen-induced hepatotoxicity 6. The platycodin D-enriched BDEs were obtained at high yield and fused with SM-loaded liposomes, resulting in suppression of hepatocyte apoptosis and the MAPK pathway. The hybrid EL combined the intrinsic anti-inflammatory properties of BDEs with the high SM loading of liposomes. BDEs also facilitated intercellular communication through endogenous biomolecules, amplifying the detoxification effects of SM without inducing cytotoxicity. Thus, plant-derived hybrid ELs show great potential as both drug carriers and active therapeutic agents.

In addition to mitigating hepatotoxicity, hybrid ELs have also been applied to treat liver fibrosis. Zhang et al. developed hybrid ELs composed of BMSC-derived exosomes and hydroxychloroquine (HCQ)-loaded vitamin A (VA, retinol)-modified liposomes (HCQ@VA-Lip-Exo) 100 (Figure 3A). BMSC-derived exosomes provided innate tropism to activate aHSCs, while VA-modified liposomes enhanced targeting via retinol-binding protein, which is overexpressed on the surface of aHSCs. Accordingly, as shown in Figure 3B, HCQ@VA-Lip-Exo accumulated in aHSCs and inhibited autophagy, a key driver of fibrogenesis. Moreover, HCQ@VA-Lip-Exo minimized off-target effects. These findings highlight the potential of hybrid ELs to combine exosomal specificity with liposomal engineering to achieve efficient and safe chemotherapeutic delivery.

### 5.2. Peptide delivery

Oral therapeutic peptide delivery is hindered by rapid enzymatic degradation and poor gastrointestinal permeability 101,102. Hybrid ELs offer innovative strategies to overcome these obstacles. Xiao et al. prepared semaglutide (SET)-loaded milk exosome -liposome nanovesicles (mExos@DSPE-Hyd-PMPC) designed to navigate sequential gastrointestinal barriers 103 (Figure 4A). Milk exosomes contributed membrane proteins that resisted enzymatic degradation, while liposomes improved SET loading efficiency. The hybrid EL mExos@DSPE-Hyd-PMPC incorporated pH-responsive zwitterionic polymers enabling adaptive surface properties: a hydrophilic, mucus-penetrating state in the jejunal lumen and a charge-reversed, cell-adhesive state at the mildly acidic epithelial surface. This design facilitated efficient traversal of the mucus barrier. Upon reaching jejunal epithelial cells, the membrane proteins and positively charged surfaces of mExos@DSPE-Hyd-PMPC enabled efficient transport across apical, intracellular, and basolateral barriers. Consequently, mExos@DSPE-Hyd-PMPC significantly improved SET oral bioavailability (Figure 4B). These findings demonstrated the ability of hybrid ELs to overcome limitations of oral peptide delivery, including protease degradation and poor mucosal permeability.

### 5.3. RNA delivery

Both exosomes and liposomes have been used for RNA delivery, but each has limitations: exosomes exhibit low loading efficiency for anionic RNA, while liposomes lack targeting ability for RNA delivery 104,105. Hybrid ELs have emerged as a promising strategy to overcome these limitations. Pre-encapsulation of RNA into liposomes enhances entrapment efficiency compared with direct exosomal electroporation, while preservation of exosomal surface proteins facilitates cell-/organ-selective targeting 106,107.

Xie et al. fabricated a hybrid EL (siRNA-HEV) using DNA zipper-mediated membrane fusion between corneal epithelium-derived exosomes and siRNA-loaded liposomes for treating dry eye disease (DED) 46 (Figure 5A). siRNA-HEV retained exosomal homing capabilities and improved siRNA loading efficiency of liposomes. By avoiding destructive methods, siRNA-HEV kept exosomal integrity. siRNA-HEV showed enhanced gene silencing and effectively alleviated inflammation in a DED mouse model. In another study using siRNA-loaded hybrid ELs, Jhan et al. prepared a hybrid EL by co-extruding tumor-derived exosomes with liposomes, and loading siRNA by electroporation 92. The hybrid EL promoted effective gene silencing, compared with commercial reagents. In addition, the hybrid EL enabled scalable production and kept exosomal targeting. This example highlighted the potential of hybrid ELs for RNAi therapeutics in cancer treatment.

In another study, Evers et al. developed siRNA-loaded hybrid ELs that activated endothelial signaling and migration, demonstrating cell-type-dependent uptake and gene-silencing efficacy 108. Furthermore, hybrid ELs retained exosome-mediated biological functions, such as endothelial cell activation, and improved biocompatibility relative to conventional liposomes.

Hu et al. engineered C-X-C motif chemokine receptor 4 (CXCR4)-overexpressing NIH-3T3 exosomes, which were co-extruded with antagomir-188-loaded liposomes to treat age-related bone loss 109. The hybrid ELs targeted bone marrow via stromal cell-derived factor 1 (SDF1) -CXCR4 interactions, facilitated osteogenic differentiation, suppressed adipogenesis in BMSCs, reversed bone loss, and reduced cortical bone porosity in an age-related osteoporosis mouse model. For colorectal cancer (CRC), folic acid (FA)-functionalized hybrid ELs (ELNPs) enhanced tumor-specific targeting via folate receptor-mediated uptake, and delivered ALKBH5 mRNA to modulate cancer-associated metabolic pathways 110 (Figure 5B). ELNPs restored tumor-suppressive gene expression, reshaped the tumor microenvironment by inhibiting glycolysis, and achieved potent inhibitory effects in preclinical colorectal tumor models. Furthermore, the FA-modified hybrid ELs enhanced RNA stability, facilitated precise targeting, and tackled intricate disease mechanisms through multimodal therapy. Collectively, hybrid ELs address critical challenges in RNA delivery, including stability, specificity, and functional integration.

### 5.4. DNA delivery

Hybrid ELs also enable targeted and safe delivery of DNA cargoes. Tong et al. developed Lipo@HEV, a novel tumor-derived antigen-loaded hybrid EL, combining immunostimulatory bacterial outer membrane vesicles (OMVs) with cationic liposomes 70 (Figure 6A). Lipo@HEV promoted dendritic cell maturation, activated cytotoxic T-cells, and exhibited efficient lymph node trafficking and deep tissue penetration, highlighting its ability to bypass physiological barriers.

CRISPR/Cas9 gene editing requires cell-specific delivery to minimize systemic toxicity 111-114. Hybrid ELs address this challenge by integrating exosomal tropism with liposomal plasmid-loading. As a paradigm, Liang and co-workers engineered CRISPR/Cas9 plasmid-loaded hybrid ELs modified with a CAP peptide and matrix metalloproteinase 13 (MMP13), enabling penetration of dense cartilage matrices in arthritic rats and selectively delivering plasmids to chondrocytes 115. The hybrid ELs suppressed MMP-13 expression and attenuated cartilage degradation.

Lin et al. used membrane fusion to prepare hybrid ELs for CRISPR/Cas9 delivery to MSCs, which are typically resistant to liposomal transfection 116. The hybrid ELs retained exosomal surface proteins, facilitating MSC uptake, while liposomal components protected CRISPR/Cas9 plasmid integrity. The system successfully eliminated β-catenin in human MSCs, demonstrating its ability to overcome cellular resistance mechanisms.

Yaghoobi et al. developed ErbB4-targeted hybrid ELs for hypoxia-sensitive CRISPR/dCas9 delivery to prevent placental insufficiency 10 (Figure 6B). Hybrid ELs encapsulated S/MAR episomal vectors with hypoxia-responsive promoters, upregulated microRNA-30d in trophoblasts, enhanced angiogenesis and implantation, and achieved trophoblast-specific uptake without affecting the inner cell mass. ErbB4-targeted hybrid ELs crossed the placental barrier and demonstrated potential for spatiotemporal gene editing control. These findings highlight the ability of hybrid ELs to combine exosomal biological advantages with liposomal engineering flexibility to facilitate precise CRISPR/Cas9 delivery across physiological barriers.

### 5.5. Co-delivery

Because both exosomes and liposomes possess lipid bilayers capable of encapsulating hydrophilic or lipophilic cargoes, hybrid ELs acquire this dual loading capability 91,117,118. This co-loading capacity of hybrid ELs facilitates combination therapies. Hybrid ELs combine exosomal biocompatibility and innate targeting with liposomal tunability and high loading capacity, promoting targeted co-delivery to diseased tissues.

As a paradigm, Zhu et al. fabricated hybrid ELs (Lip-CExo@PTX) co-loaded with paclitaxel and cytotoxic granules, based on CAR-T-derived exosomes and lung-targeted liposomes to treat systemic malignancies 119 (Figure 7A). As displayed in Figure 7B-C, liposomes mediated primary lung accumulation through charge-directed tropism, while single-chain variable fragments (scFvs) from CAR-T-derived exosomes enabled secondary tumor-specific targeting (Figure 7D). Lip-CExo@PTX co-delivered paclitaxel and cytotoxic granules, and simultaneously engaged in PD-L1 blockade. This multi-modal activity caused chemotherapy-induced immunogenic cell death, T cell-mediated toxicity, and checkpoint inhibition (Figure 7E-G). Thus, Lip-CExo@PTX addressed pharmacokinetic limitations of free drugs and resistance mechanisms within the tumor microenvironment.

Hybrid ELs have also been applied to co-deliver chemotherapeutics for the targeted treatment of cancer. Zhou et al. developed IHEL, a hybrid EL co-loaded with IR780 and hemin, using exosomes derived from M1-polarized RAW264.7 cells and liposomes, for targeted melanoma therapy 120. IHEL exploited exosomal tumor-homing properties, enabling selective accumulation in melanoma tissues. Under NIR laser irradiation, IHEL demonstrated superior tumor elimination due to the combined photodynamic/photothermal therapy and immunomodulation via ferroptosis activation, glycolytic suppression, and the repolarization of tumor-associated macrophages toward an M1 phenotype. IHEL thus represents a promising photo-immunotherapy strategy for melanoma.

For glioma treatment, Wang et al. developed pHybrid/SAB-CPT, a hybrid EL co-loaded with cryptotanshinone (CPT) and salvianolic acid B (SAB), using blood cell-derived exosomes and tLyp-1-modified liposomes. pHybrid/SAB-CPT leveraged exosomal transferrin receptors for BBB penetration and tLyp-1-modified targeting for enhanced tumor parenchyma penetration via neuropilin-1 interactions 121. This dual-targeting strategy enabled efficient co-delivery of CPT and SAB to glioma. pHybrid/SAB-CPT maintained exosomal surface proteins for physiological barrier navigation and incorporated liposomal components for controlled release and tumor targeting. Blood cell-derived exosomes contributed to immune evasion, while liposomes provided tunable pharmacokinetics.

Hybrid ELs can also co-deliver RNA cargoes and chemotherapeutics for brain-targeted glioma therapy. Ma et al. fabricated a brain-targeted hybrid EL (PMRT) by co-incubating macrophage-derived exosomes with thermo-sensitive liposomes, to co-deliver temozolomide (TMZ) and siRNAs (si-mTOR and si-c-Myc) for glioma treatment 71. PMRT utilized exosomal surface proteins, such as integrins, to enhance BBB penetration, while thermo-sensitive liposomes provided stable encapsulation and controlled release of TMZ and siRNAs. PMRT effectively reduced glioma stem cell (GSC) viability, inhibited tumorsphere formation, and synergistically suppressed GSCs by inhibiting the mTOR-c-Myc signaling pathway, thereby reversing TMZ-resistance in glioma. In orthotopic glioma xenograft mice, PMRT significantly suppressed tumor burden and prolonged survival, demonstrating strong therapeutic potential. In orthotopic glioma xenograft mice, PMRT significantly suppressed tumor growth and prolonged the lifespan of mice, highlighting the promising potential of PMRT for the efficient management of glioma.

In another study, Li et al. developed miR497/TP-HENPs, a hybrid EL combining tumor-derived exosomes with cRGD-modified liposomes to overcome chemoresistance 122. The hybrid EL co-delivered the small-molecule therapeutic drug triptolide (TP) and a tumor-suppressing microRNA (miR497) (Figure 8). Dual tumor-targeting was achieved through exosomal homologous tropism and cRGD-mediated integrin receptor recognition. miR497/TP-HENPs simultaneously blocked chemoresistance-associated signaling pathways and modulated the immunosuppressive tumor microenvironment. In addition, pH-responsive cargo release enabled spatiotemporal drug activation within an acidic tumor microenvironment. TP enhanced miR497 efficacy by depleting intracellular antioxidants, while miR497 amplified TP sensitivity to tumor cells by downregulating survival pathways. The hybrid EL exemplified multimodal therapies against complex resistance mechanisms. Hybrid ELs have also been explored for neurodegenerative diseases.

Jiang et al. developed BBB-penetrating hybrid ELs to co-deliver BACE1 siRNA (siBACE1) and TREM2 plasmid (pTREM2) for AD 123 (Figure 9A). Hybrid ELs combined MSC-derived exosomes with ROS-responsive liposomes. As displayed in Figure 9B-C, the exosomal membrane proteins facilitated neuronal targeting and lysosomal escape, while the liposomes enabled ROS-triggered release of cargoes (Figure 9D). This sequential delivery allowed initial siRNA-mediated inhibition of Aβ production followed by sustained plasmid-driven enhancement of microglial phagocytosis. The dual-gene therapeutic strategy could simultaneously target multiple pathological hallmarks, thereby establishing a self-perpetuating therapeutic cycle. As shown in Figure 9E-F, *in vivo* results indicated that hybrid ELs reduced Aβ burden, alleviated neuroinflammation, and improved microglial clearance, demonstrating strong therapeutic potential for AD.

## 6. Limitations and challenges

Hybrid ELs offer promising potential to treat various diseases, yet some challenges hinder clinical translation.

### 6.1. Stability

Though hybrid ELs are generally more stable than exosomes, they remain susceptible to physiological fluctuations, like temperature, pH, and ROS, which can destroy structural integrity and destabilize cargo 124. Dynamic *in vivo* physiological variables may reduce therapeutic effect, underscoring the need for long-term *in vivo* stability studies.

### 6.2. Immunogenicity

While the immunogenicity of exosomes is low due to their endogenous origin, the introduction of liposomes may alter immune responses 24. For example, PEGylated liposomes can induce anti-PEG antibodies, triggering ABC 125. Additionally, heterologous exosome sources may provoke immune responses 126. Thus, rigorous immunogenicity is essential to ensure the efficacy of hybrid ELs.

### 6.3. Clearance and Biodegradation

Ensuring biodegradation of hybrid ELs and their cargoes is critical to avoid chronic toxicity. Clearance pathways must be thoroughly studied.

### 6.4. Interspecies gap

Most hybrid EL studies rely on murine models, limiting translational relevance. Patient-derived organoids and organ-on-a-chip offer physiologically relevant human surrogates for evaluating *in vivo* PK, PD, and immunogenicity.

### 6.5. Industrialization

Exosomal heterogeneity complicates batch-to-batch reproducibility and therapeutic consistency 127. Exosomes vary with donor cell and extraction methods, affecting hybrid EL homogeneity. Scalability remains challenging due to limited yields from current isolation techniques and the difficulty of balancing regulatory frameworks for hybrid EL biosafety and characterization, which are underdeveloped, contributing to limited industrial expertise, which in turn could engender further potential delays in commercialization 24,128.

## 7. Conclusion and future directions

Future development of hybrid ELs depends on several actionable advances. Tangential flow filtration (TFF) enables large-scale exosome purification with high yield and low impurity and is already used for bulk production 129. High-throughput microfluidic platforms allow single-step, continuous-flow isolation of exosomes, outperforming conventional exosome preparation methods such as ultracentrifugation 130,131. Together, large-scale TFF and microfluidics production can reduce costs while enabling high-throughput, controlled fabrication of hybrid ELs.

Current optimization of hybrid ELs, including lipid compositions, exosome-to-liposome ratios, and fusion conditions, remains empirical. Artificial intelligence-driven high-throughput screening, including METiS technologies for lipid nanoparticle design optimization, could systematically map design-efficacy relationships, and thus accelerate the development of optimal hybrid ELs from years to months 132. Also, regulatory agencies must develop parallel frameworks to evaluate *in vivo* biodistribution, long-term stability, and immunogenicity of hybrid ELs, ensuring therapeutic efficacy and biosafety. By integrating these advances, hybrid ELs have the promising potential to overcome the limitations of exosomes and liposomes and pioneer next-generation precision therapies for the treatment of various diseases.

## Author contributions

**Jian Yu:** Writing-Original draft preparation.** Lujiao Yu:** Writing-Reviewing and Editing.** Fei Ren:** Writing-Reviewing and Editing. **Kai Cui:** Supervision. **Gongping Sun:** Conceptualization. **Hong Pan:** Supervision. All authors have read and approved the final manuscript.

## Figures and Tables

**Figure 1:**
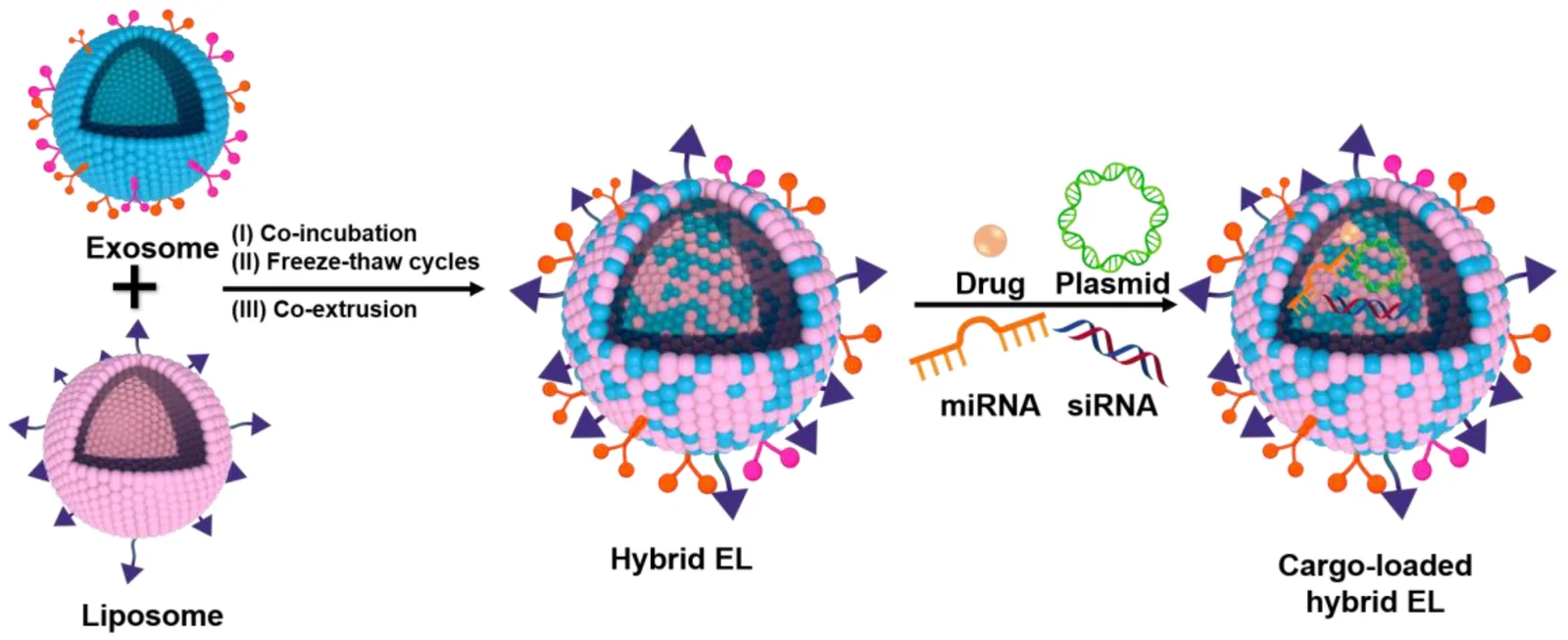
Schematic illustration of the fabrication and cargo loading of hybrid exosome-liposome (EL). There are three main strategies for the fabrication of hybrid EL: the co-incubation method, the freeze-thaw method, and the co-extrusion method. Diverse cargos can be loaded into the hybrid EL, including chemotherapeutic drugs, miRNAs, siRNAs, and plasmids.

**Figure 2:**
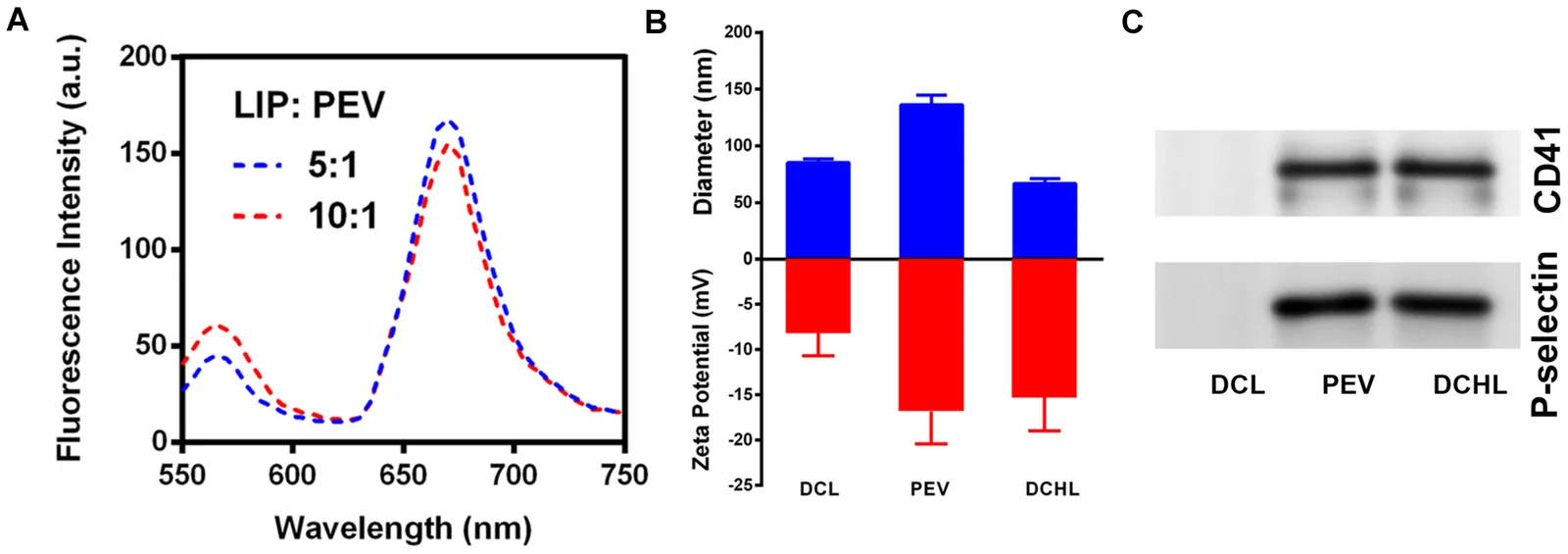
Verification of the membrane fusion structures of hybrid EL prepared by co-extrusion. (A) The membrane of platelet-derived exosomes labeled with fluorescent dyes and fused with a growing number of liposomes, indicating the FRET effect. (B) The hydrodynamic diameter and zeta potential of different formulations. All data are presented as the mean ± SD (n = 3). (C) The markers of platelet-derived exosomes, including CD41 and P-selectin, were detected using Western blotting. Adapted with permission from 91, copyright 2023 Cell Press.

**Figure 3:**
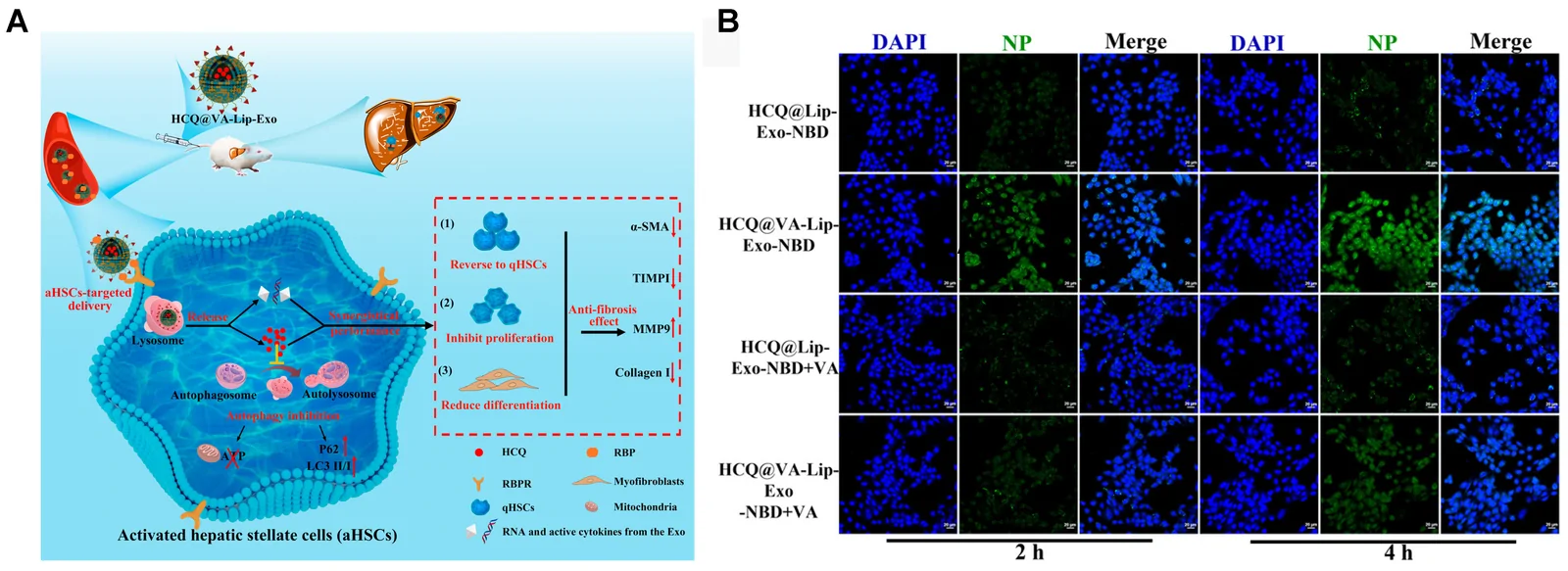
(A) Schematic illustration of HCQ@VA-Lip-Exo for aHSC-targeted autophagy inhibition and synergistic treatment of liver fibrosis. (B) Targeting uptake of HCQ@VA-Lip-Exo by aHSCs (Scale bar = 20 μm). Adapted with permission from 100, copyright 2023 American Chemical Society.

**Figure 4:**
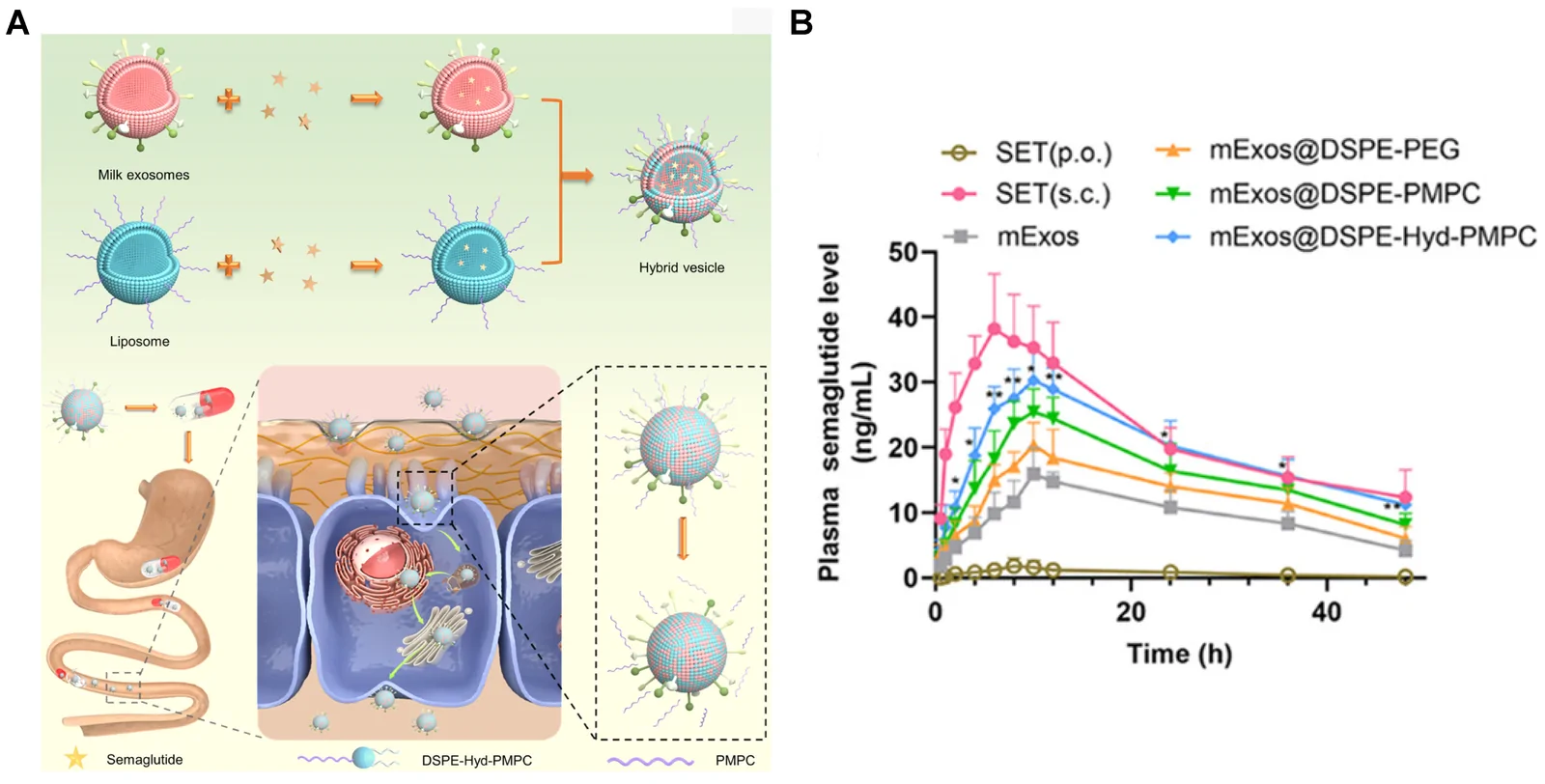
(A) Schematic illustration of mExos@DSPE-Hyd-PMPC for oral delivery of semaglutide. (B) Plasma semaglutide concentration-time profiles in rats. All data are presented as the mean ± SD (n = 5). SET (p.o.): oral semaglutide solution group; SET (s.c.): subcutaneous injection semaglutide solution group. *p < 0.05, and **p < 0.01 versus the mExos group. Adapted with permission from 103, copyright 2024 American Chemical Society.

**Figure 5:**
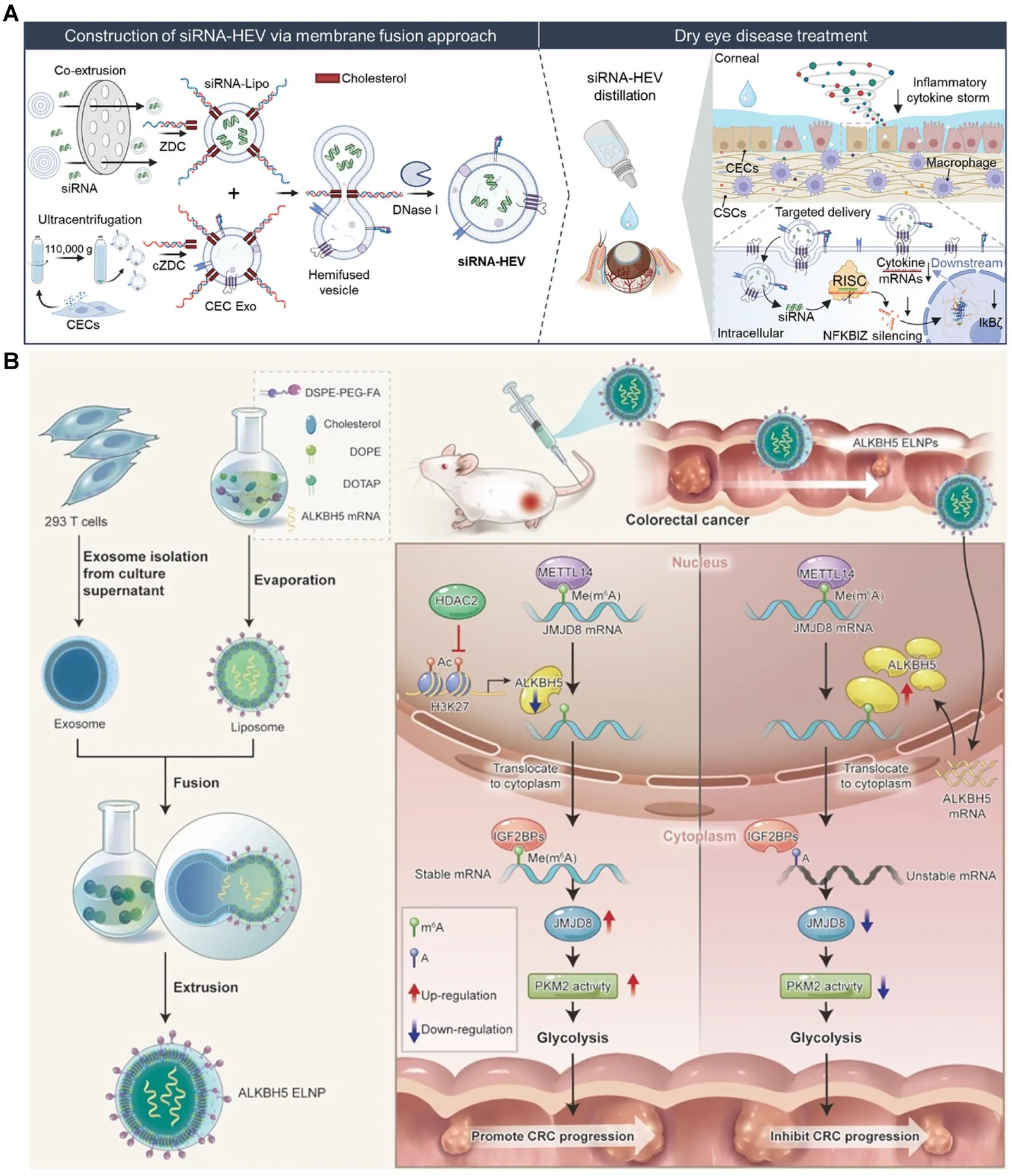
(A) Schematic illustration of the membrane fusion strategy to load anti-NFKBIZ siRNA into HEV construct for the treatment of dry eye disease 46. Copyright 2024, John Wiley and Sons. (B) Schematic illustration of the ALKBH5-mediated regulatory network in colorectal cancer progression, and ALKBH5-ELNP mediated ALKBH5 mRNA delivery inhibits colorectal tumorigenesis. Adapted with permission from 110, copyright 2023 American Chemical Society.

**Figure 6:**
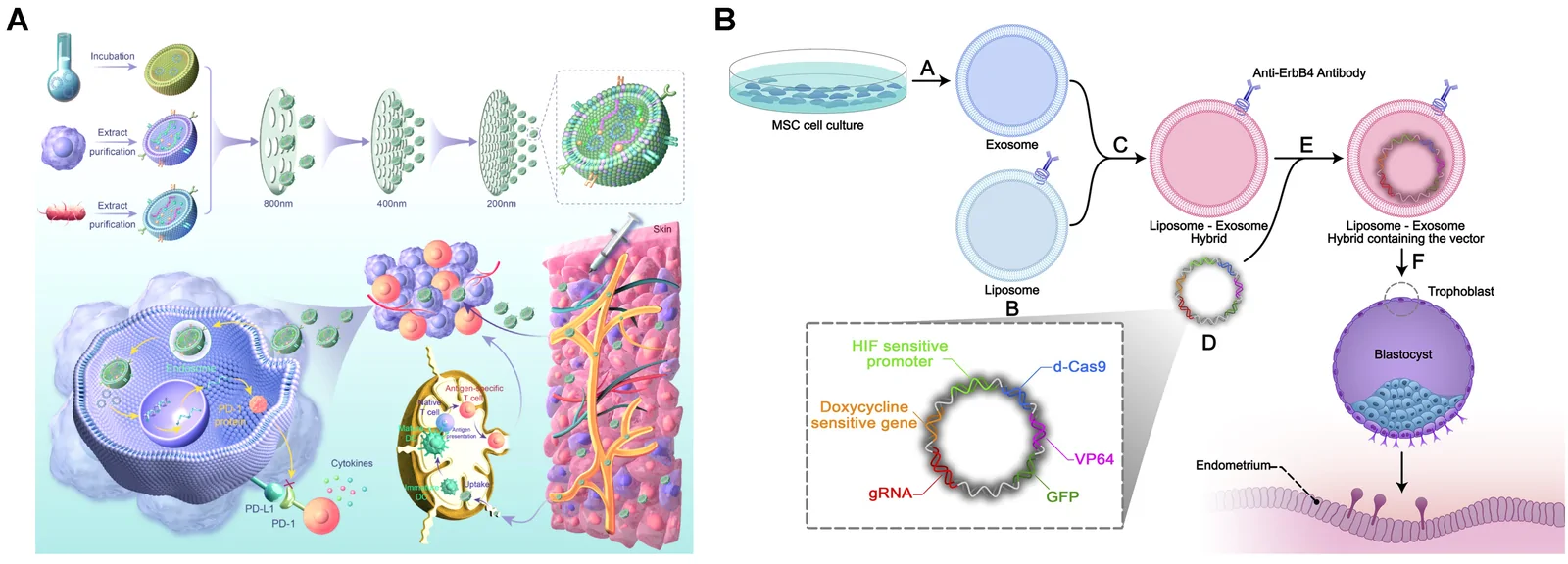
(A) Schematic illustration of Lipo-PD-L1@HEV for synergistic cancer immunotherapy. Lipo-PD-L1@HEV was fabricated by fusing extracellular vesicles with a plasmid-loaded liposome. Upon peritumoral injection, Lipo-PD-L1@HEV can localize in tumor for PD-L1 blockade, and penetrate into lymph node to efficiently initiate dendritic cell, antigen presentation and cytotoxic T cell activation, synergistically inhibiting the tumor 70. Reproduced under terms of the CC BY license. Copyright 2023, The Authors, published by Elsevier. (B) Schematic illustration of hypoxia-sensitive ErbB4-targeted hybrid EL for CRISPR/dCas9 delivery system. A: Isolation of MSC exosomes. B: Antibodies against ErbB4 conjugated on the surface of liposomes. C: The hybrid EL will be fabricated by co-incubating MSC-derived exosomes and ErbB4-targeted liposomes. D: Non-viral S/MAR episomal vector will be constructed. E: Electroporation will be used to encapsulate the designed episomal S/MAR vector into the hybrid EL. F: The hybrid EL will be added to the trophoblast cell media, and the episomal vector will specifically enter the trophoblast. Adapted with permission from 10, copyright 2023 Frontiers.

**Figure 7:**
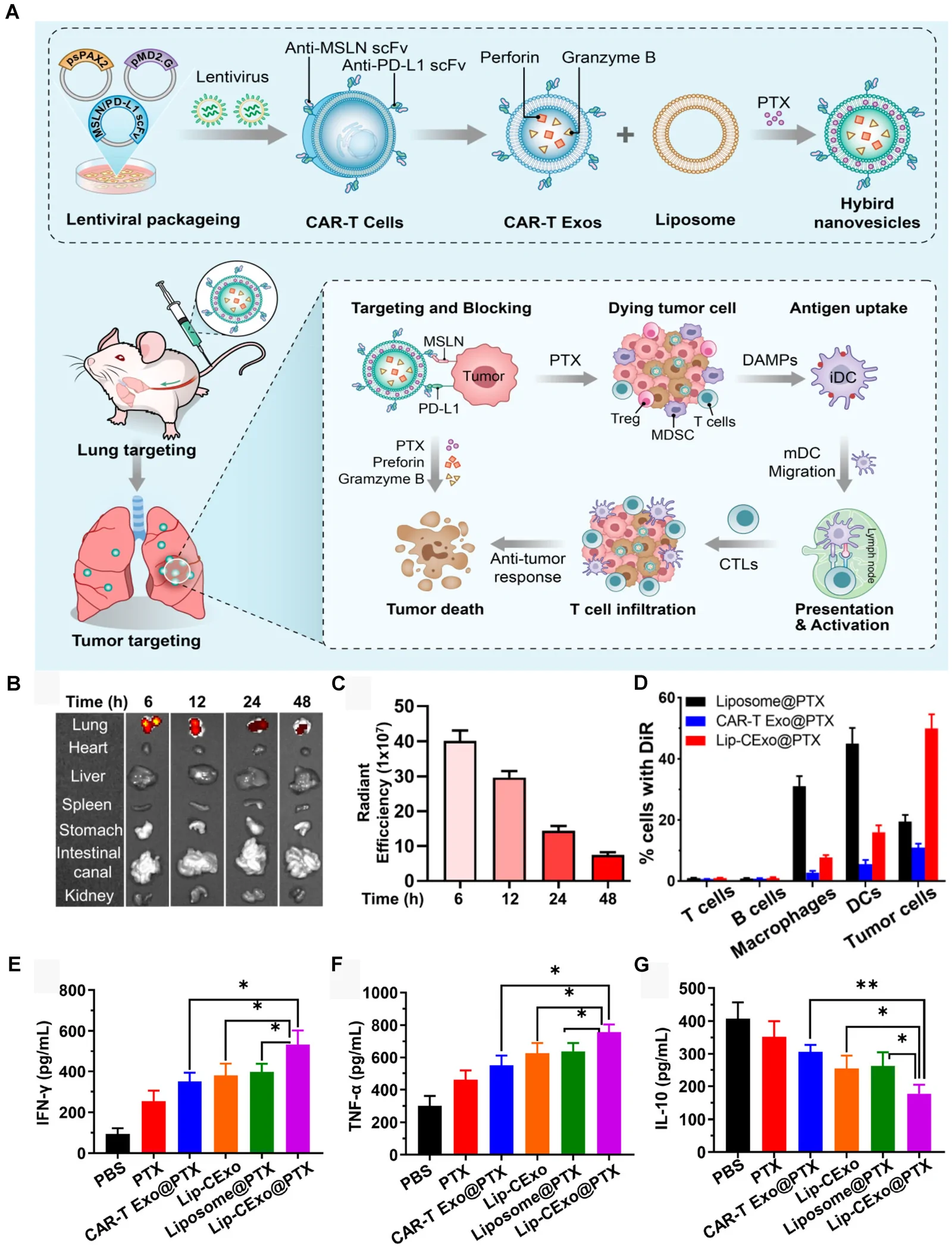
(A) Schematic illustration of Lip-CExo@PTX for lung cancer therapy. (B,C) Following injection of DiR-labeled Lip-CExo, the fluorescence of the major organs was analyzed by the IVIS system *ex vivo* at different time points. (D) The fluorescence of different cells in the tumor microenvironment 8 h post-injection of DiR-labeled CAR Exos, liposomes, or Lip-CExos was analyzed by flow cytometry. (E-G) The concentrations of IFN-γ, TNF-α, and IL-10 in tumor tissues were measured by ELISA after treatment with different drugs. All data are presented as the mean ± SD (n = 3). *p < 0.05, and **p < 0.01. Adapted with permission from 119, copyright 2023 American Chemical Society.

**Figure 8:**
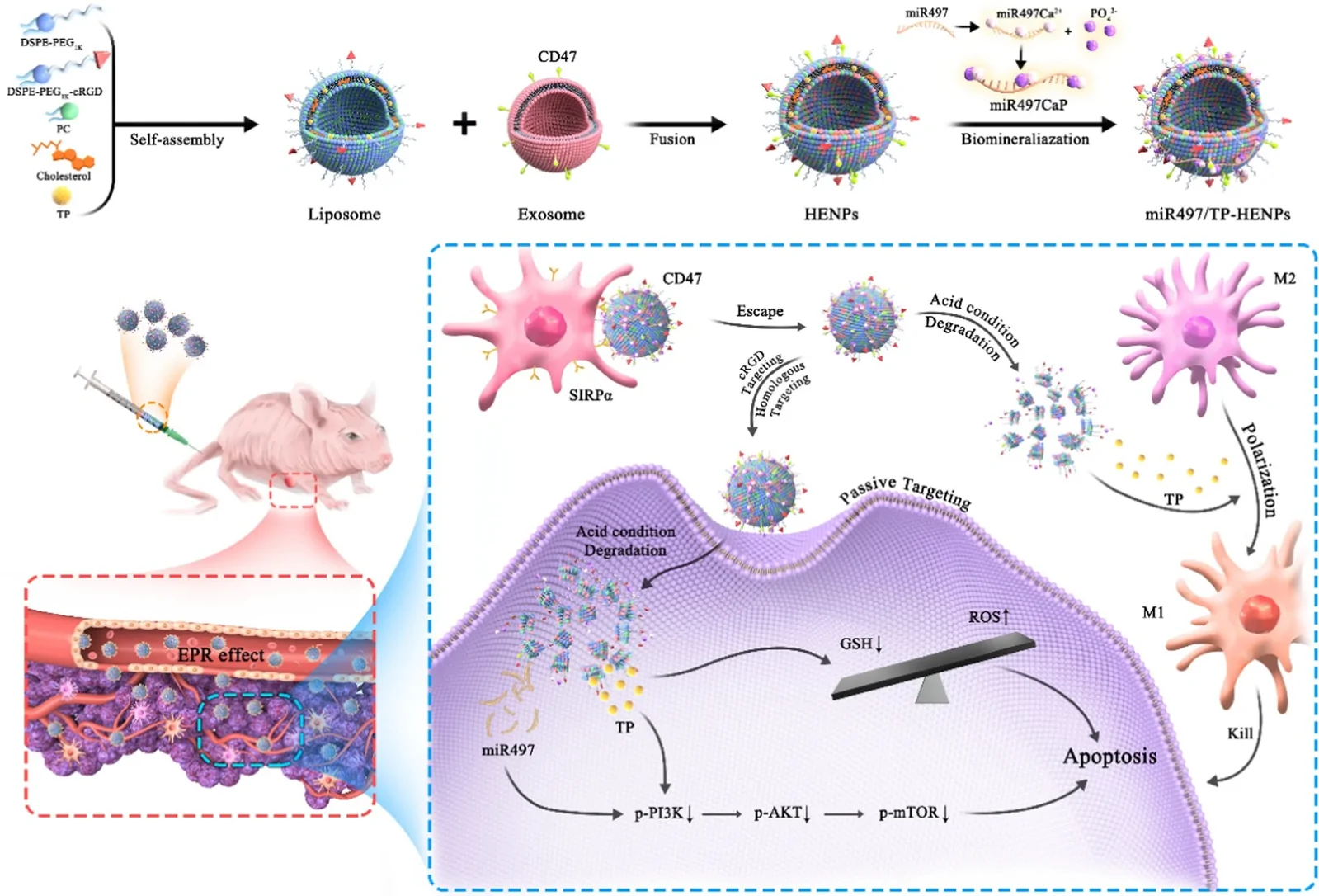
Schematic illustration of the preparation process and therapeutic mechanism of miR497/TP-HENPs. When miR497/TP-HENPs reach OC cells, miR497 and TP synergistically inhibited the PI3K/AKT/mTOR pathway. TP stimulated ROS production, and TP modulated polarization of M2 macrophages into M1 macrophages, synergistically overcoming OC resistance. Adapted with permission from 122, copyright 2022 BMC Press.

**Figure 9:**
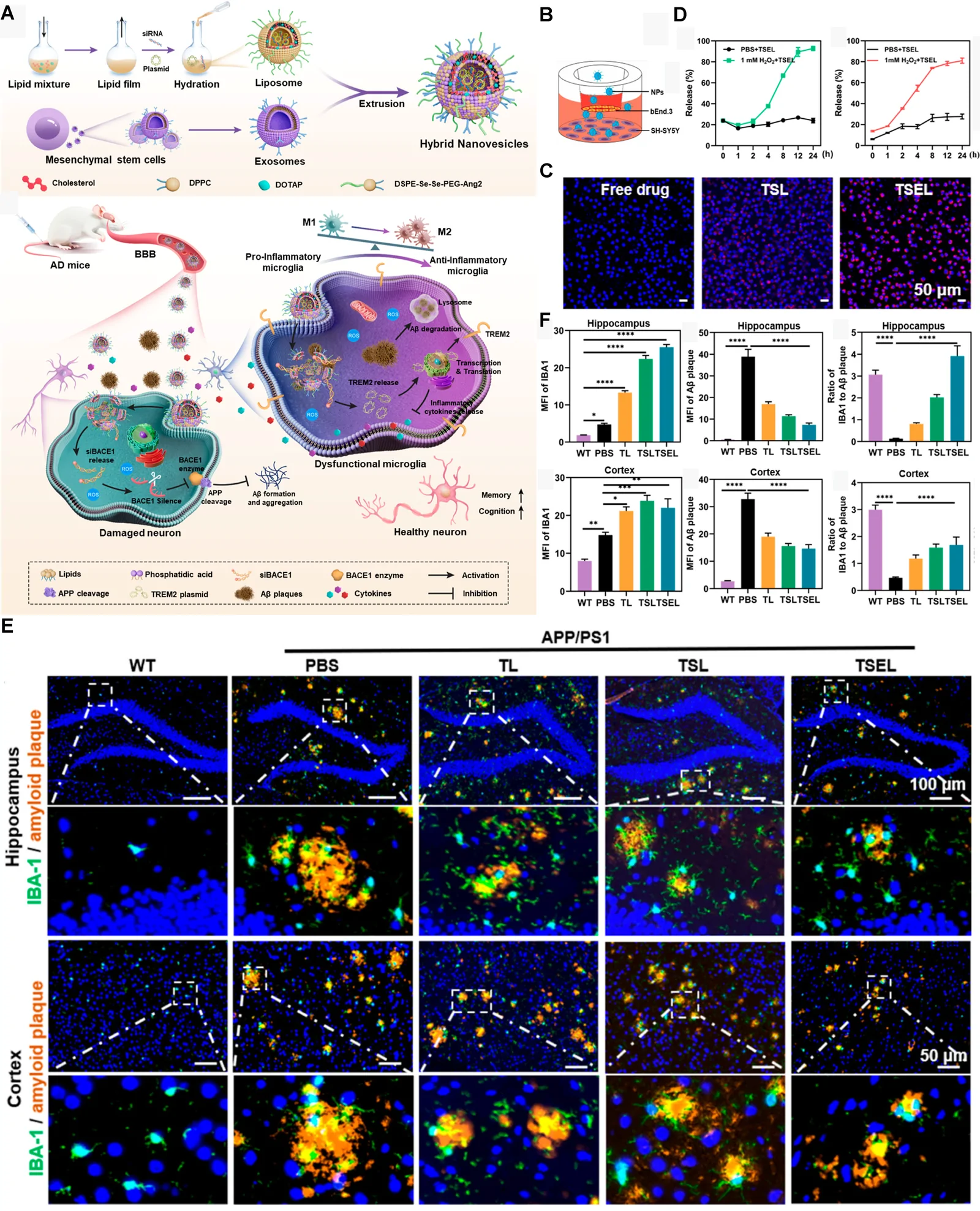
(A) Schematic illustration of the preparation process and therapeutic mechanism of TSEL. (B) Schematic diagram of the *in vitro* BBB model. (C) CLSM images of Cy5-siRNA fluorescence signal to evaluate the BBB penetration capability of TSEL (Scale bar = 50 μm). (D) The cumulative release of pTREM2 and siRNA from the TSEL with or without 1 mM H_2_O_2_. All data are presented as the mean ± SD (n = 3). (E) Representative immunofluorescence images of amyloid plaque (orange) with microglia (green) colocalization in the hippocampus (Scale bar = 100 μm) and cortex (Scale bar = 50 μm) of AD mice treated with PBS, TL, TSL, or TSEL. The bottom row of each image displays an enlarged version of the white boxes in the upper image. (F) Quantification analysis of Aβ intensity and the number of IBA1-positive microglia engulfing Aβ in the hippocampus and cortex of AD mice. All data are presented as the mean ± SD (n = 3). *p < 0.05, **p < 0.01, and ***p < 0.001. Adapted with permission from 123, copyright 2024 American Chemical Society.

**Table 1:** Comparisons of exosomes from different sources.

Source	Advantages	Disadvantages	Examples
Body fluid	Easy collection	Batch differences	Plasma, semen, urine, saliva, ascites fluid, lavage fluid, cerebrospinal fluid, bile, lymph
Mammalian cell	Steady batch production	High large-scale production cost	MSC, macrophage, dendritic cell, B cell, T cell, platelet, epithelial cell, neuron
Edible plant	Inherent anti-inflammatory effect	Relative low yield	Ginger, garlic, grapefruit, carrot, broccoli
Edible food	Scalable and safe	Batch differences	Milk, strawberry, blueberry, grape, apple, lemon

**Table 2:** Comparisons of characteristics between different fabrication strategies for hybrid EL.

Category	Co-incubation method	Freeze-thaw method	Co-extrusion method
Mechanism of fusion	Passive intermolecular interactions	Exosomes and liposomes breakdown into smaller nanovesicles because of freeze-thaw cycles	Lipid bilayer rearrangement and reassembly to decrease the free energy
Advantages	Ease to prepare without any additional operational steps Negligible impact on cargos and exosomal membrane	High fusion efficiency	Controllable particle size and size distribution Short fabrication time
Disadvantages	Limits in fusion efficiency Heterogeneous size distribution and poor stability	Cargo leakage susceptibility Potential impairment of surface protein integrity and function	Low entrapment efficiency for hydrophilic drugs Potential impairment of surface protein integrity and function
Fusion efficiency	Low	High	Medium
Surface protein of exosome	Almost no impact	Potential impairment of surface protein bioactivity	Potential impairment of surface protein integrity and function
Stability	Low	Medium	High

## Abbreviations

aHSC: activate hepatic stellate cell
ABC: accelerated blood clearance
AD: Alzheimer’s disease
ALIX: Alg2-interacting protein X
BBB: blood-brain barrier
BDEs: balloon flower root-derived exosome-like nanoparticles
BMSC: bone marrow mesenchymal stem cell
BRB: blood-retinal barrier
COX IV: cytochrome c oxidase subunit IV
CPT: cryptotanshinone
CRC: colorectal cancer
cRGD: cyclic arginyl-glycyl-aspartic acid
CXCR4: C-X-C motif chemokine receptor 4
DED: dry eye disease
DOX: doxorubicin
FA: folic acid
FRET: Forster resonance energy transfer
GSC: glioma stem cell
HCQ: hydroxychloroquine
ICD: immunogenic cell death
MAPK: mitogen-activated protein kinase
mExo: milk exosome
MISEV2018: minimal information for studies of extracellular vesicles 2018
MMP13: matrix metalloproteinase 13
MPS: mononuclear phagocyte system
MSC: mesenchymal stem cell
NPs: nanoparticles
OMVs: outer membrane vesicles
PD: pharmacodynamics
PEG: polyethylene glycol
PK: pharmacokinetics
RGC: retinal ganglion cell
ROS: reactive oxygen species
SAB: salvianolic acid B
SDF1: stromal cell-derived factor 1
SET: semaglutide
scFvs: single-chain variable fragments
SM: silymarin
TFF: tangential flow filtration
TMZ: temozolomide
TP: triptolide
TSG101: tumor susceptibility gene 101
UCNPs: upconversion nanoparticles
VA: vitamin A
